# Risk factors associated with the colonization of group b streptococcus during pregnancy: preliminary results

**DOI:** 10.1186/1472-6955-14-S1-S5

**Published:** 2015-10-08

**Authors:** Lidia Francés Ribera, Àngels Paulí Cabezas, Paula Amorós Ferrer, Montserrat Villanueva Guevara, Avelina Tortosa Moreno, M Victoria Cambredó Aparicio

**Affiliations:** 1 School of Nursing at the University of Barcelona, Barcelona, Spain; 2 Sexual and Reproductive Health Program La Riera Badalona of the Catalan Institute of Health, Spain; 3 Sexual and Reproductive Health Program Sabadell of the Catalan Institute of Health, Spain

## Background

In Spain, the rate of colonization with GBS in pregnant women is 11%-18.2% and in Catalonia between 13-16%. The vertical transmission rate is 50%, and in the absence of avoidance manoeuvres, between 1-2% of new-borns develop GBS infection, which is the main bacterial agent in neonatal sepsis precocious.

## Aim

To analyse the impact of risk factors for GBS colonization during pregnancy in Catalonia.

## Methods

An analytical case-control study. Data collection took place in different primary health care centres, in the Sexual and Reproductive Health Program (Badalona and Sabadell Units) of the Catalan Health Institute. Pregnant women attending the midwife clinics for prenatal care during the first trimester. The case group included pregnant women who presented positive culture for GBS at 35-37 weeks and the control group included those who had negative culture. The data collection period was from January 2013 to April 2015.

## Results

The preliminary results we are presenting belong to the Sexual and Reproductive Health Care Unit of Badalona. The studied sample was 403 pregnant women. 89 of them were considered losses from the study. Of origin 72.8% were autochthonous and 27.2% were foreign. It was more frequent to be CASE being foreign than autochthonous (p=0.014) OR=2.3. 5.7% had a history of GBS positive culture in a previous pregnancy. It was more frequent to be CASE when there was positive GBS history in a previous pregnancy (p=0.04) OR=5.7. About depositional habits, 56.6% defecate once per day. It was more frequent to be CASE when there was more than one defecation per day (p < 0.001) OR=3.5. As for genital hygiene, 48.4% performed genital hygiene once per day. It was more frequent to be CASE when the genital hygiene was performed two or more times per day (p=0.006) OR=2.7.

## Conclusions

The risk factors for the GBS colonization are: positive culture during the first trimester, being foreign, positive GBS history in previous pregnancies, the number of defecations per day and the number of times per day that genital hygiene is performed. The identification of risk factors for GBS colonization will make it possible to identify the ones that are potentially modifiable and will aid the design of preventive interventions for midwives and improve access to the health of pregnant women and new-borns.

Project funded by:

– Research Commission of the School of Nursing at the University of Barcelona.

*– Official College of Nurses of Barcelona. PR-2708-12*.

